# Characterization of thymus-associated lymphoid depletion in bovine calves acutely or persistently infected with bovine viral diarrhea virus 1, bovine viral diarrhea virus 2 or HoBi-like pestivirus

**DOI:** 10.1007/s00705-017-3523-x

**Published:** 2017-08-09

**Authors:** Shollie M. Falkenberg, Fernando V. Bauermann, Julia F. Ridpath

**Affiliations:** 1 Ruminant Disease and Immunology Research Unit, National Animal Disease Center, United States Department of Agriculture, Agricultural Research Service, Ames, IA 50010 USA; 2 Veterinary and Biomedical Sciences, South Dakota State University, Brookings, SD USA

## Abstract

Naïve pregnant cattle exposed to pestiviruses between 40-125 days of gestation can give birth to persistently infected (PI) calves. Clinical presentation and survivability, in PI cattle, is highly variable even with the same pestivirus strain whereas the clinical presentation in acute infections is more uniform with severity of symptoms being primarily a function of virulence of the infecting virus. The aim of this study was to compare thymic depletion, as measured by comparing the area of the thymic cortex to the medulla (corticomedullary ratio), in acute and persistent infections of the same pestivirus isolate. The same general trends were observed with each pestivirus isolate. Thymic depletion was observed in both acutely and persistently infected calves. The average thymic depletion observed in acutely infected calves was greater than that in age matched PI calves. PI calves, regardless of infecting virus, revealed a greater variability in amount of depletion compared to acutely infected calves. A trend was observed between survivability and depletion of the thymus, with PI calves surviving less than 5 weeks having lower corticomedullary ratios and greater depletion. This is the first study to compare PI and acutely infected calves with the same isolates as well as to evaluate PI calves based on survivability. Further, this study identified a quantifiable phenotype associated with potential survivability.

## Introduction

Bovine viral diarrhea virus 1 (BVDV-1) and BVDV-2, along with classical swine fever virus (CSFV) and border disease virus (BDV) are taxonomically classifiable within the *Pestivirus* genus of the family *Flaviviridae* [28]. In addition, several unassigned atypical pestiviruses have been characterized and proposed to be representatives of new species; including giraffe pestivirus, antelope pestivirus, HoBi-like pestivirus (also reported as a putative BVDV-3) and Bungowannah virus [5, 18, 19, 31]. Clinical signs resulting from HoBi-like pestivirus infection of cattle are indistinguishable from those that result from infection with either BVDV-1 or BVDV-2 [9–13]. Upon infection, the severity of the clinical disease in naïve immune competent animals can range from mild/subclinical to severe, depending on the virulence of the strain involved in the infection [16]. Acute infection with noncytopathic (ncp) BVDV is characterized in the periphery by a transient lymphopenia following exposure that can be observed by day 3, with the greatest lymphopenia occurring between 6-9 days post-infection (dpi) and recovery to baseline by 12-14 dpi [15]. The viral distribution in lymph nodes is evident on day 6 with clearance by day 13 [21]. Comparisons of acute infection of calves exposed to a typical or high virulent BVDV or a HoBi-like pestivirus have been reported and all 3 viruses resulted in reduced numbers of circulating white blood cells (WBCs) typically observed during acute pestiviral infections [26]. Following transplacental infection between 40-125 days of gestation, ncp BVDV and HoBi-like pestivirus strains can induce a state of viral immunotolerance that leads to lifelong viral persistence in the immunologically tolerant animal [2, 22]. Not only does the interaction of the ncp BVDV persisting strain subvert the adaptive immune system, but also subverts various aspects of the innate immune system [23]. While the BVDV persistently infected (PI) animal does not respond to the virus it carries, it does have the capability to normally respond to other antigens and produce antibodies (Ab) following infection with BVDV strains that are antigenically divergent from the virus the animal is harboring [7, 22, 29, 35]. Another interesting characteristic of BVDV and HoBi-like pestivirus PI animals is the variation in the genetic diversity or breadth of viral swarms [25, 33] observed between individual animals.

The differences observed in the survival rate of PI animals infected with BVDV could be due to variation in the complex interaction of mechanisms that support immunotolerance and maintenance of the viral swarm that are specific to each individual animal. In comparison to persistent infections, the interplay of mechanisms involved in the immune response to acute infection is relatively simple and variations in clinical response are more likely due to variations in virulence or biotype of the infecting virus. Previous work has demonstrated the immunological consequences of highly virulent (HV) and typical virulent (TV) acute infections on the immune system and lymphoid tissues, reporting as great as an 80% and 50% reduction in the gross weight of the thymus for naïve calves infected with HV and TV BVDV, respectively [15]. Grossly, thymic depletion can be visually described as the thymus having lobes that are thin and lack the robust lobular characteristics of normal thymic tissue that is demonstrated by a reduction in gross weight. This reduction in gross weight can histologically be characterized as depletion of the cortex and the lack of discernible transition between the cortex and medulla. The depletion of the cortex can be quantified in paraffin embedded tissues stained with hematoxylin and eosin (H&E) using the color deconvolution morphometric analysis and comparison of the corticomedullary ratio [15] with Spectrum ImageScope digital pathology analysis software (Aperio Technologies, Vista, CA, USA).

The goal of this study was to determine if histological depletion patterns observed in the thymus correlated with clinical presentations. To this end, we compared the degree of thymus depletion in calves acutely infected with BVDV or HoBi-like pestivirus strains to PI calves infected with the same strain or a pestivirus strain with similar virulence.

## Materials and methods

### Viruses and exposure to viruses

Two ncp field strains belonging to two different BVDV species were used; ncp BVDV type 2 strain RS886 (BVDV-2-RS886) and ncp BVDV type 1b strain TGAN (BVDV-1b-TGAN). The ncp HoBi-like pestivirus strains used were HoBi_D32/00 and Italy-1/10-1. Both BVDV-2-RS886 and BVDV-1b-TGAN were isolated at the NADC (United States) from clinically normal PI calves [8, 21]. Two different ncp HoBi-like pestivirus strains were also used, HoBi_D32/00 was isolated at the Friedrich-Loeffler-Institut (Germany) as a contaminant in a fetal bovine serum (FBS) lot that originated from South America [27], while the isolate Italy-1/10-1 was identified in an outbreak of respiratory disease in an Italian herd [9]. All pestivirus strains were propagated in bovine turbinate cells that had been tested and were found to be free of BVDV and HoBi-like pestivirus [6]. Cells were grown in complete cell culture medium composed of minimal essential media (MEM; Sigma-Aldrich, St. Louis, MO), supplemented with L-glutamine (1.4 mM; Gibco, Life Technologies, Grand Island, NY), 1% of antibiotic-antimycotic-100X (Invitrogen, Life Technologies, Carlsbad, CA), and 10% fetal bovine serum (FBS; PAA, Ontario, Canada) that was heat inactivated. Fetal bovine serum was tested and found to be free of BVDV and HoBi-like pestivirus strains and antibodies against BVDV or HoBi-like pestivirus [6]. Pestiviral titers for each respective virus were determined via dilution on bovine turbinate cells [4]. Endpoints were verified by immunoperoxidase staining based on the monoclonal antibody N2 that bound to an epitope in the E2 protein of the bovine pestiviruses used [5, 24]. A total of 5 ml of inoculum of each respective pestivirus-infected cell culture lysate (2.5 ml/nostril; ~10^6^ TCID/ml) was delivered directly into the nasal passages for all the inoculated animals.

### Study design

#### Persistently infected calves

Seventeen crossbred heifers, approximately 10-11 months of age, that were negative for bovine pestivirus antigen and antibodies as previously described [6, 26] were segregated into 4 groups; BVDV-1-TGAN (*n* = 3), BVDV-2-RS886 (*n* = 6), HoBi-like pestivirus HoBi_D32/00 (*n* = 4), and Hobi-like pestivirus Italy-1/10-1 (*n* = 4). Due to space limitations these groups were infected sequentially rather than simultaneously. All heifers were artificial inseminated (AI) following estrous synchronization and were exposed to a bull for approximately 45 days after AI to achieve a uniform group of animals at similar gestational periods. Pregnancy status was confirmed by both ultrasound and a commercially available enzyme-linked immunosorbent assay (ELISA; Bio-PRYN™, Biotracking LLC, Moscow, ID) that measures the amount of pregnancy-specific protein B in serum. All pregnant heifers were moved to either animal biosafety level (ABSL) 2 (BVDV-1 and -2) or BSL3-Ag (HoBi-like pestivirus) containment at approximately 55 days of gestation and housed in individual pens throughout the duration of the study. The methods for generation of the HoBi-like pestivirus PI calves were described previously [2].

Heifers were inoculated on approximately day 70 of gestation and blood was collected on day 6-post infection for virus detection by RT-PCR in the buffy coat [2, 32]. At approximately days 100 and 160 of gestation, blood was again collected from the heifers and serum was submitted to confirm heifers were still pregnant using the previously described ELISA. The heifers were induced approximately 10 days prior to their estimated due date and the resulting calves were isolated from the dam at birth to ensure they were colostrum deprived. Surviving calves were kept in ABSL2 (BVDV strains) or BSL3-Ag (HoBi-like pestivirus strains) containment in individual crates for the duration of the study as previously described [20]. All surviving calves were euthanized after day 35 (>5 weeks of age) with a pentobarbital sodium injection (Fatal Plus, Vortech Pharmaceutical Ltd., Dearborn, MI) at 1 ml/4.54 kg and thymus tissue was excised.

#### Acutely infected calves

Twenty-four colostrum-deprived Holstein bull calves approximately 3 weeks of age that tested negative for BVDV antibodies and antigen were used for the acute infection phase. Due to the space limitations groups of calves infected with different viruses were infected sequentially rather than simultaneously. Calves deprived of colostrum were procured within 4 hours of birth from the same dairy herd, tested and housed as described previously [20, 26]. The pestivirus strains used in the generation of PI calf section used for acute infection inoculation were; BVDV-2-RS886 strain (*n* = 6), HoBi_D32/00 strain (*n* = 6), Italy-1/10-1 strain (*n* = 6) and a group of control calves (*n* = 6) were mock inoculated with 5 ml (2.5 ml/nostril) of lysate prepared from non-infected tissue culture cells. Each treatment group was housed separately in the appropriate biocontainment facilities (ABSL2 for BVDV and BSL3-Ag for HoBi-like pestivirus). Calves were monitored daily, euthanized with a pentobarbital sodium injection (Fatal Plus, Vortech Pharmaceutical Ltd., Dearborn, MI) at 1 ml/4.54 kg and thymus tissue was excised on day 14 post infection (~5 weeks of age).

#### Tissues, histology and morphometric analysis

The cervical thymus was excised and immediately fixed by immersion in 10% neutral buffered formalin for microscopic evaluation followed by processing and embedding in paraffin as described previously [20]. Paraffin embedded tissues were cut in 5-μm sections, stained with H&E and examined by light microscopy and subjected to morphometric analysis using a Scan Scope XT workstation digital pathology scanner (Aperio Technologies, Vista, CA). Images were scanned in at a 20X magnification, visualized at 1X and digitized with Spectrum ImageScope Software digital pathology analyzer (Aperio Technologies, Vista, CA, USA). The digitized images were analyzed using a modified standard color deconvolution analysis algorithm as previously described [15]. Rather than using a grid system to evaluate multiple fields within each section of tissue, the digital pathology software allowed annotation and analysis of an entire tissue section. The section analyzed measured a minimum of 5 mm^2^ total area (equivalent to 25 fields of 0.2 mm^2^) and a maximum of 50 mm^2^. This size range allowed the analysis of entire lobes of normal and depleted thymus tissue. The positive color channel as designated by the Aperio system was hematoxylin (1) and the threshold values modified from the standard algorithm for pixel intensity included: the black threshold (0, clear glass), weak positive threshold (185), medium positive threshold (105) and strong positive threshold (105) in the digital pathology software, with the threshold range from 0-255 pixel intensity. Threshold values are standardized based on H&E stained tissues from control animals from the same experiment. Ten lobes from 2 control animals were used to determine the threshold values using the algorithm tuning tool to differentiate the cortex and medulla based on color intensity. Based on these parameters, detection of strong positive staining indicated cortex and detection of weak positive staining indicated medulla. Quantification of the corticomedullary ratio was based on the ratio of strong positive to weak positive values. Variability of the strong:weak ratio was calculated using the variability function in Microsoft Excel, 2013 (Microsoft Corporation, Redmond, WA).

## Results

Initially comparisons were made between acute and PI infections for each viral isolate. Due to the similarity in trends, subsequent comparisons were made between all acutely infected animals regardless of infecting virus and all PI animals regardless of infecting virus.

Briefly, as previously described [2, 3], all inoculated heifers producing a PI calf were viremic on day 6 post-inoculation and virus neutralization titers at parturition ranged from 1144 to 9153 to viruses from the same species as the inoculum. The outcome of calves infected *in utero* is summarized in Table 1 and as previously described [2, 3]. Of the 6 heifers infected with BVDV-2-RS886, one heifer aborted (fetus was collected) and three gave birth to calves that survived less than 5 weeks and two gave birth to calves that survived the full 5 weeks of the experiment. All of the three heifers infected with BVDV-1b-TGAN, gave birth to calves that survived the full five weeks of the experiment. Of the four heifers infected with HoBi_D32/00, two gave birth to calves that survived the duration of the experiment, one aborted (fetus was collected) and one lost the pregnancy without visible signs of abortion (no fetus collected). Of the four heifers infected with Italy-1/10-1, two gave birth to calves that did not survive to five weeks and two gave birth to calves that survived the duration of the experiment. All calves in the acutely infected groups as well as the control group survived up to 5 weeks of age.

**Table 1:** Number of calves for each respective type of infection as well as the viral isolate used, categorized by survivability age groups

Infection	Viral isolate	# Calves >5 weeks age	# Calves <5 weeks of age	Fetus	Aborted	Total
PI	BVDV-2-RS886	2	3	1	0	6
	BVDV-1b-TGAN	3	0	0	0	3
	HoBi_D32/00	2	0	1	1	4
	Italy-1/10-1	2	2	0	0	4
Acute	BVDV-2-RS886	6	N/A	N/A	N/A	6
	HoBi_D32/00	6	N/A	N/A	N/A	6
	Italy-1/10-1	6	N/A	N/A	N/A	6
Control	Mock cell lysate	6	N/A	N/A	N/A	6

**Fig. 1:**
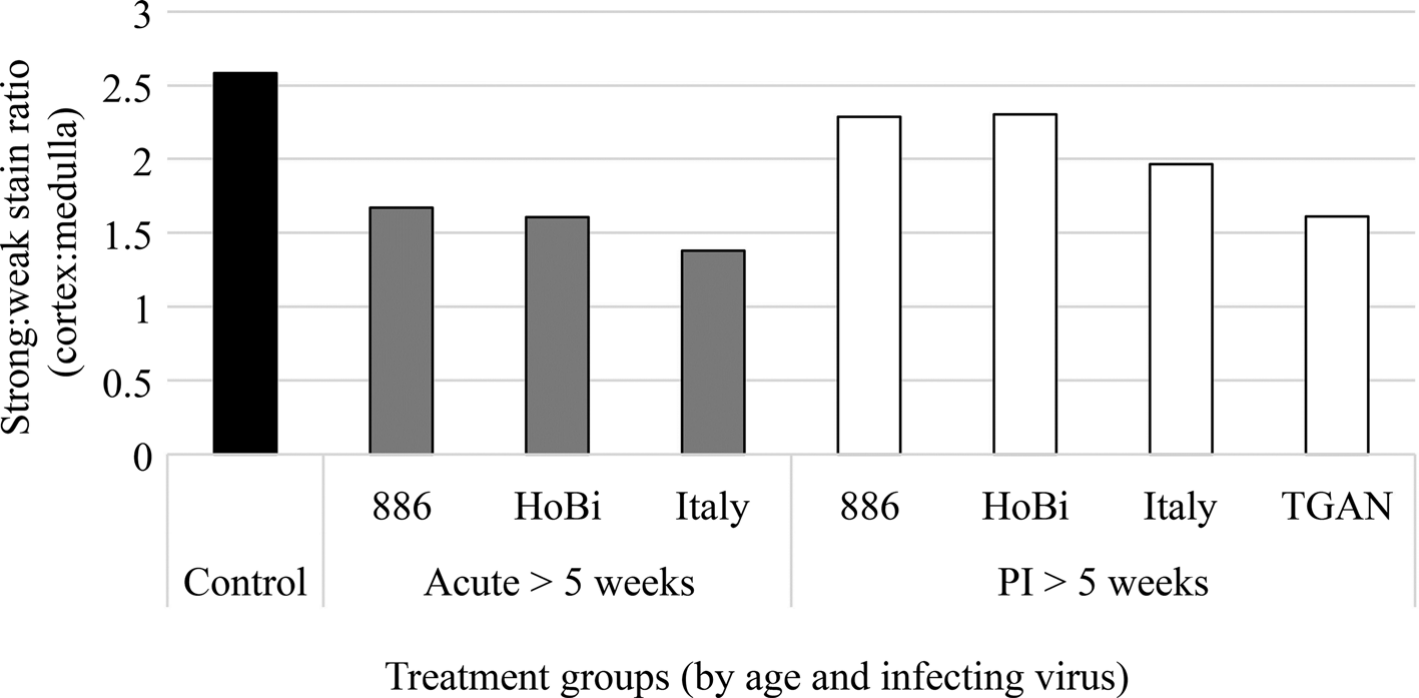
Strong positive and weak positive staining representing the corticomedullary ratio, respectively. Data represent means for the thymus section analyzed from the following groups, acutely or persistently infected (PI): control, BVDV-2-RS886 (886), BVDV-1b-TGAN, HoBi_D32/00 (HoBi) and Italy-1/10-1 (Italy). Tissue samples were collected when all calves were at a minimum of 5 weeks of age (>5 weeks)

Treatment groups representing each pestivirus for both acute and PI calves as well as control calves that were at least 5 weeks of age are represented in Fig. 1. As stated in the preceding section, the strong:weak stain ratio represents the ratio of cortex to medulla (corticomedullary ratio). Control calves had the greatest (2.58) corticomedullary ratio as compared to both acute and PI age matched calves. In general, acutely infected calves (harvested 2 weeks after infection; 5-8 weeks of age), had lower corticomedullary ratio than PI calves that survived for the duration of the experiment (>5 weeks of age). While this trend was observed regardless of infecting virus, there was variation in the extent of thymic depletion that correlated with the viral strain. The ratios observed in calves infected either acutely or persistently with Italy-1/10-1 were comparatively lower while the ratios observed in calves acutely or persistently infected with BVDV-2-RS886 were comparatively higher.

**Fig. 2:**
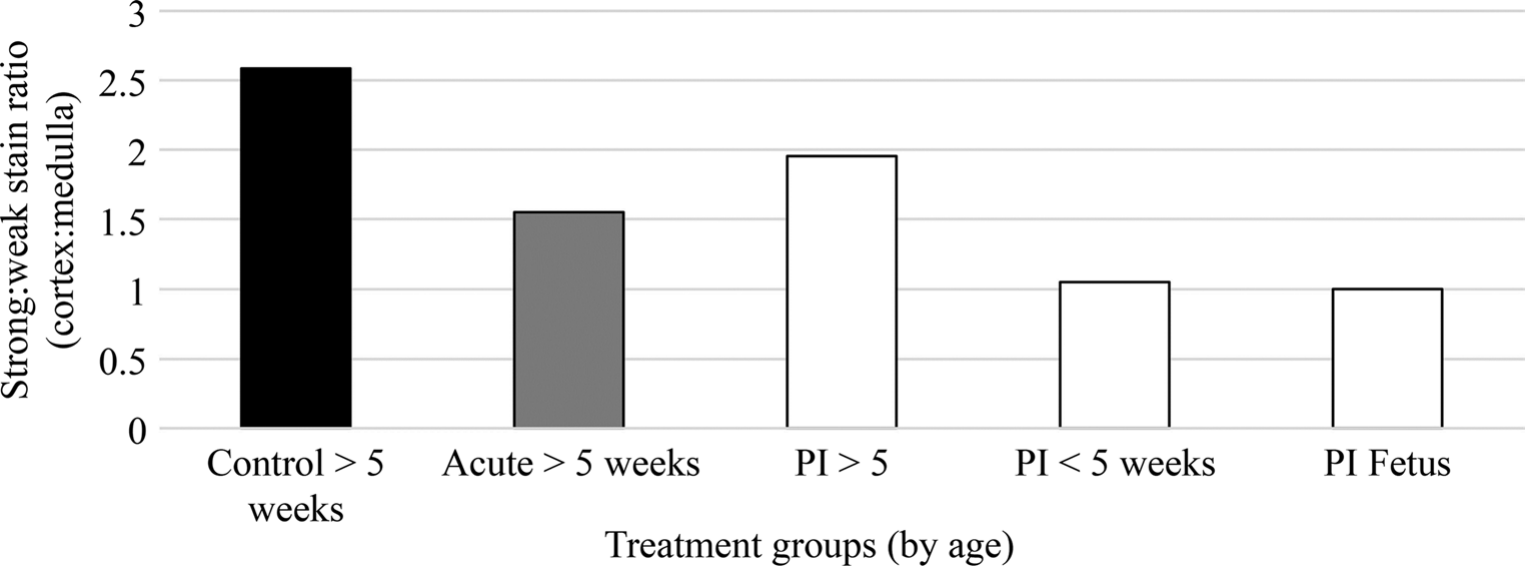
Strong positive and weak positive staining representing the corticomedullary ratios, respectively. Data represent means for the thymus section analyzed from the following groups, acutely or persistently infected (PI): control, BVDV-2-RS886 (886), BVDV-1b-TGAN, HoBi_D32/00 (HoBi) and Italy-1/10-1 (Italy). Tissue samples were collected when calves were euthanized and subsequently categorized into age ranges

**Fig. 3:**
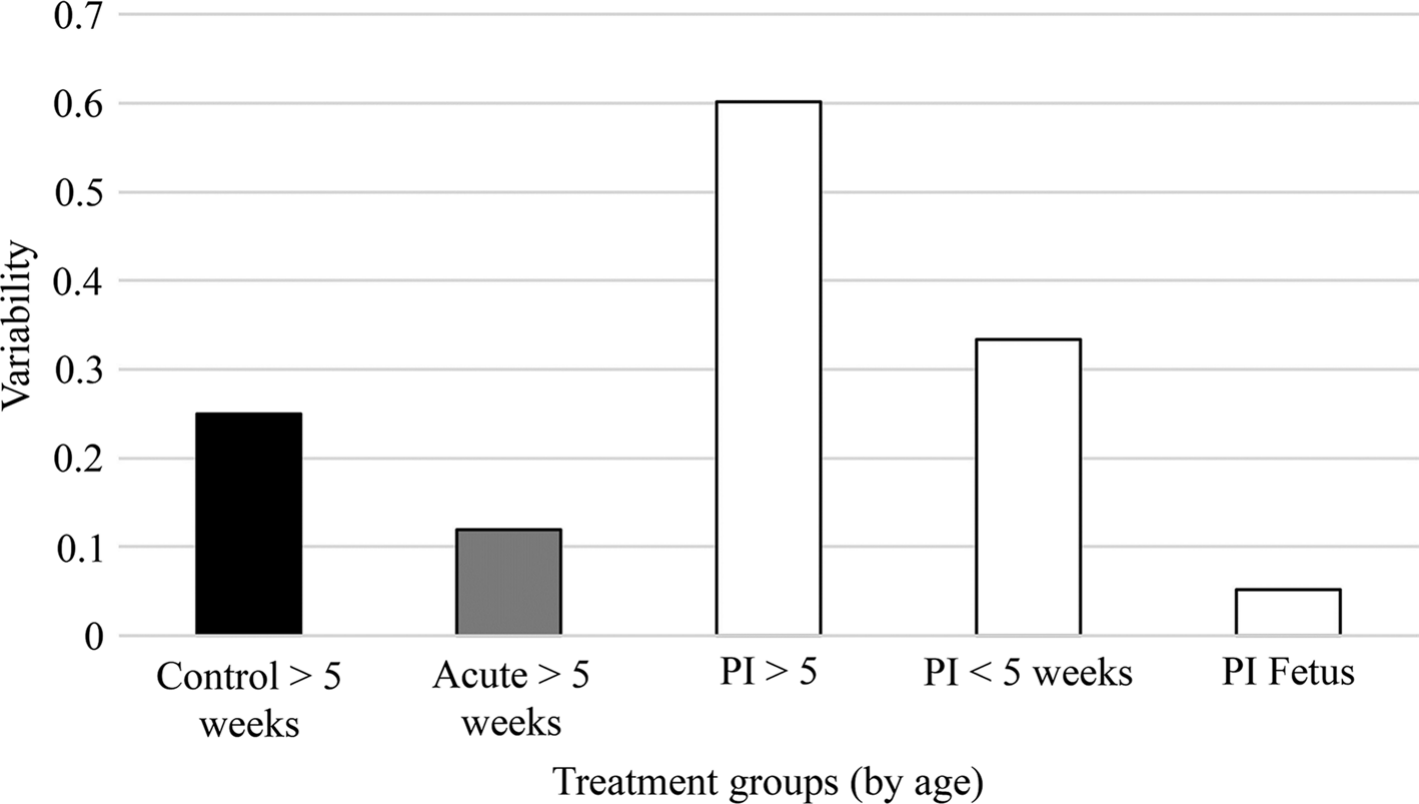
Mean variability calculations for the strong positive and weak positive staining ratio for the thymus sections analyzed from the following groups, acutely or persistently infected (PI): control, BVDV-2-RS886 (886), BVDV-1b-TGAN, HoBi_D32/00 (HoBi) and Italy-1/10-1 (Italy). Tissue samples were collected when calves were euthanized and subsequently categorized into age ranges

While PI calves that survived the duration of the study had higher corticomedullary ratios than calves harvested 2 weeks after an acute infection, this was not true of either PI calves that did not survive the duration of the experiment or aborted fetuses. Calves were grouped into acute infection, PI > 5 weeks of age, PI < 5 weeks of age, and aborted PI fetuses to compare corticomedullary ratio (Fig. 2). In general, acutely infected and PI calves that survived the duration of the study had ratios approximately 40 percent and 25 percent lower than control calves, respectively. The ratios of PI calves that did not survive the duration of the experiment and aborted fetuses were approximately 60 percent lower than that of control calves and approximately 50 percent lower than PI calves that survived the duration of the study (Fig. 3). While minor differences were observed between groups acutely infected with different pestivirus strains, the variability among calves infected with the same virus was low. This suggests that the degree of thymic depletion observed in acute infections is dependent on viral strain. In contrast, the variability in the corticomedullary ratio of PI animals was greatest within groups of calves carrying the same virus as between groups of calves carrying different viruses. The degree of variability correlated with the survival of the PI calf rather than the viral isolate. The lack of variability was most pronounced in aborted fetuses and calves that did not survive the duration of the experiment. These results would suggest that the degree of thymic depletion is linked to survivability.

**Fig. 4:**
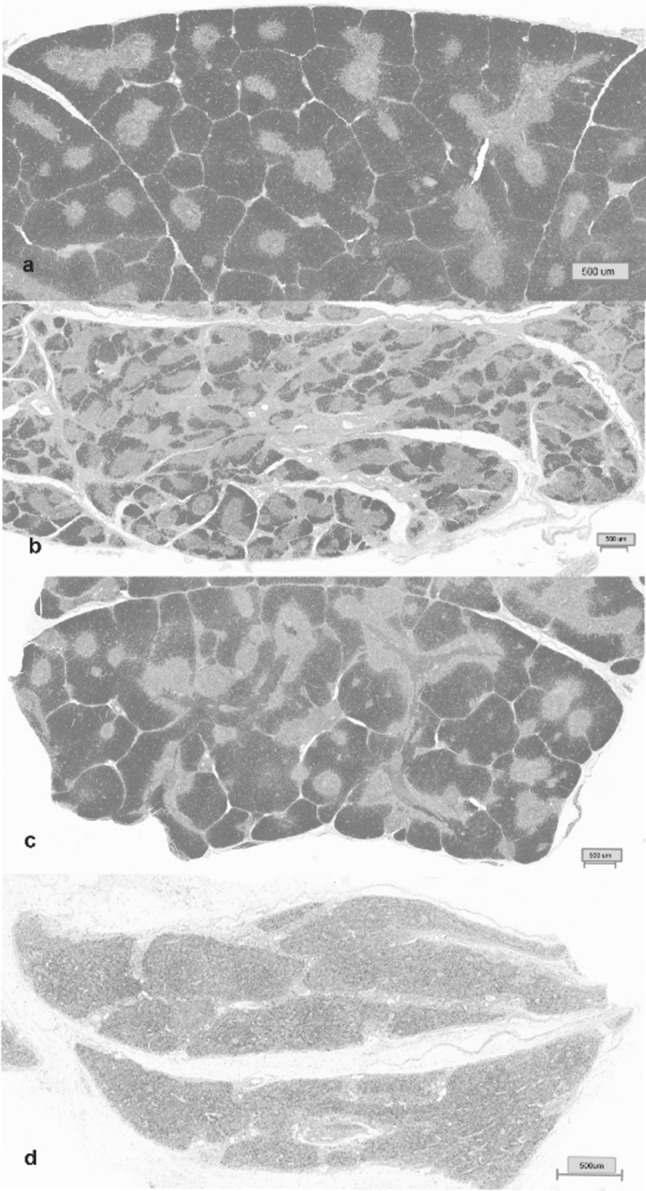
Section of tissue from the thymus of a: (a) control calf, (b) calf acutely infected, (c) a clinical PI calf and (d) clinical PI calf. Cortical atrophy of the thymus was most pronounced in clinical PI calves, followed by calves that were acutely infected. Control and a clinical PI calves had similar thymic patterns. Hematoxylin and eosin staining. Bar = 500 μm

The variability associated with persistent infections described in the previous section is represented in Fig. 4. The variability is associated with two distinct patterns observed in H&E thymic sections from PI calves. The two patterns consistently observed included; the lack of distinct and discernible borders between the thymic cortex and medulla was consistently observed in PI calves that did not survive for the duration of the study, whereas sections from PI calves that survived the duration of the study had, more consistently, distinct transitions between the cortex and medulla (Fig. 4). Of the nine PI calves surviving greater than 5 weeks, six calves had distinct cortex and medulla transitions similar to that of control calves and only three calves surviving the duration had cortex and medulla transitions similar to that of animals not surviving the duration of the study, as shown in Fig. 4d.

## Discussion

This is one of the first studies to compare the effects of BVDV and HoBi-like pestivirus acute and persistent infections on thymic morphology. While minimal differences were observed between viral strains and type of infection, all of the pestiviruses used in this study have similar effects on immune tissue, similar clinical presentations, with lesions that are indistinguishable. The data support that infection with bovine pestiviruses, in naïve pregnant cattle, can lead to a variety of outcomes including rebreeding (infertility), abortions, mummification, stillbirth, congenital malformations, birth of weak and undersized calves and birth of PI calves [1]. Similarly, these data support that the outcome of persistent infections with pestiviruses is variable, ranging from congenital malformations, to calves with no gross abnormalities that fail to thrive (“poor doers”) to clinically normal calves that survive to adulthood [30]. Much of the literature characterizing persistent infection has been performed using PI calves that have survived the neonatal period. However, looking at “surviving PI animals” does not provide a complete picture of PI phenotypes. From an epidemiological point of view the surviving population is of concern because this group is a major source of bovine pestivirus transmission. However, focusing on this group can result in missing a large piece of the puzzle that surrounds fetal infections. Studying PI fetuses and calves that succumb in the first few days and weeks of life could provide insight into the factors influencing survivability.

Results from this study suggest that survivability and adverse outcomes in PI animals is linked to the severity of thymic depletion as illustrated by a lower corticomedullary ratio in aborted fetus populations and in calves that succumbed when they are less than 5 weeks of age. Similar data evaluating the size of the thymus by sonography in human fetuses, describe a relationship between small fetal thymuses and adverse perinatal outcomes [14]. Depletion of the thymus due to BVDV is generally thought to be associated with ncp BVDV infection and severity of depletion correlates with the virulence of the infecting virus [15]. RNA viruses, in particular, can mutate rapidly due to errors introduced during successive rounds of genome replication introducing antigenic variants that give rise to a greater breadth of quasispecies. While this strategy may aid the virus’s ability to evade the immune system following acute infections, a high rate of mutation could be detrimental in persistent infections. In acute infections the virus is recognized and immune cells replicating virus are destroyed. In persistent infections, the immune system does not recognize the virus and infected immune tissue is maintained. A wider range of variants generated within the viral swarm of a PI could result in a higher physiological cost to maintain the immunosuppression associated with immunotolerance. While it would appear that there is a connection between survivability and thymic depletion, the cause of the depletion is not known. Further these studies are not designed to determine if depletion was a primary cause of survivability or a consequence of other factors associated with survivability. One possibility is that thymic depletion observed in calves that succumbed prior to 5 weeks of life is related to failure to control variation within viral swarms and a resulting breakdown of immunotolerance and destruction of immune tissue. While depletion of the thymus appears to be linked with survivability, the greatest degree of variability in the corticomedullary ratio was observed in the PI calves greater than 5 weeks of age. Since these calves were euthanized after 5 weeks of age this only provides a snapshot of the variation that is observed at this age and it can only be speculated what the potential outcomes would be for these calves. It could be hypothesized that the calves with the lower corticomedullary ratio, if allowed to live their full span of life, would have had a lower survivability rate and calves with greater corticomedullary ratio could maintain immunotolerance more effectively and would have lived longer. While it has been reported that PI calves have chronic up-regulation of type 1 IFN [17] and innate immune responses as measured by an increase in cytokines in the spleen of PI animals [36] it is not understood how these measures are related to immunotolerance. Furthermore, the breadth and complexity of BVDV populations in PI cattle from a single point infection have been described and the degree of variation that was observed would suggest that there is no “one size fits all” when it comes to PI calves [25] and the current results would support this observation. Furthermore, in support of this hypothesis observations from a subset of these calves, the HoBi PI calves, suggest that the breath and complexity of the swarms were variable and calves that had greater diverse viral swarms as previously reported [33, 34] also had greater thymic depletion. This is represented in Fig. 4c and 4d that illustrate the variability in thymic depletion of two HoBi-like pestivirus PI calves that have a less diverse viral swarm (Fig. 4c) and a more diverse viral swarm (Fig. 4d). Collectively, all of these observations would suggest that there is an interplay between the viral swarms and the immune response associated with PI cattle and one of the consequence of this interplay getting out of balance could be depletion of the thymus due to destruction of immune tissue incurred in the process of eliminating variant viruses arising in the swarm. The up-regulation of type I interferon and increased cytokines could be one method to contain and maintain viral swarms but any deviation from tight control might lead to immune intolerance and subsequently death.
